# PsycEXTRA

**DOI:** 10.5195/jmla.2017.272

**Published:** 2017-10-01

**Authors:** Marilyn G. Teolis

## GENERAL DESCRIPTION

PsycEXTRA is a bibliographic database for unpublished or grey literature in psychology and the behavioral sciences. Launched in 2004, PsycEXTRA is intended to provide high-quality, searchable information to expand current knowledge in the psychological sciences. It is meant to be a companion database for PsycINFO, with no overlap between the two databases [1].

## SCOPE AND CONTENT

PsycEXTRA indexes conference abstracts, proceedings, meeting presentations, clinical trials, technical reports, patient-oriented factsheets and brochures, magazines, monographs, standards, and guidelines. Sources include research institutes, universities, governmental agencies, military agencies, associations, foundations, businesses, and other organizations. The file also includes reports and official minutes from American Psychological Association (APA) divisions. Conferences date from the early 1900s to upcoming [2]. Reports begin coverage at 1938, standards start in 1941, curricula and monographs extend to the late 1950s, and brochures and fact sheets date from 1987.

APA ensures the accuracy, timeliness, and completeness of the database’s content. Documents must be fact-based and presented in a professional format. Knowledge managers review all the content for quality, relevance, and significance. To determine whether an item is accepted, APA utilizes the following criteria: the organization’s history, its governing body, and its conventions, meetings, and publications program. Research reports must be issued by accredited schools or major labs [3].

## AUDIENCE

PsycEXTRA contains materials of interest to both health professionals and professionals in other fields. Because it focuses on content outside the peer-review system, it indexes materials before they appear in published journals and provides a single repository for grey literature from many places. Potential users include researchers, faculty, students, health care workers, and mental health professionals who are looking for the most current information. Because it serves as an archive of conference materials and monographs from the earliest days of psychology’s development as a science, the database is of value to historians. Teachers and guidance counselors helping children deal with natural disasters or traumatic events also benefit from the database’s age-appropriate standards and guidelines [4].

## INSTITUTIONAL SUBSCRIPTIONS

PsycEXTRA is available on several library database platforms, including those from EBSCO, ProQuest, and Ovid Technologies, as well as from APA’s own platform, PsycNET. The PsycNET version may be somewhat more current, since APA, as the producer, inputs records immediately into the database.

Institutional access is based on Internet protocol (IP) range. A mobile app for Apple or Android devices is free with a paid subscription. COUNTER-compliant usage statistics are available for download or SUSHI-compliant harvesting. APA provides a variety of easy-to-follow print and online training materials, including field guides, podcasts, topic guides, quick reference guides, webinars, sample records of document types, frequently asked questions, and short video tutorials.

## SEARCHING

PsycEXTRA searches can be performed in basic or advanced search modes. Allowable search terms include keywords, title, abstract, index terms (from APA’s *Thesaurus of Psychological Index Terms*), year, document type, author, and content owner. Boolean operators can be utilized for expanding or narrowing results. An important feature of the database is the ability to search by document type, especially for conferences and proceedings. I performed test searches on the PsycNET, EBSCO, ProQuest, and Ovid platforms using subject queries that demonstrate the wide range of topics covered in PsycEXTRA, including bullying, post-traumatic stress syndrome, children and disasters, and moral injury in veterans, as well as searches for various publication types (conference presentations, proceedings, and dissertations). The number of items retrieved in each of the vendors’ products was similar except for minor variations due to differences in vendor interfaces. Except for popular magazines, full text was retrievable for 70% of the items.

## CONCLUSION

Study results, white papers, dissertations, and other ephemeral materials can provide significant assistance to researchers and other people looking for the most up-to-date information. These items may appear in the grey literature as much as twelve to eighteen months before being published in peer-reviewed journals or appearing in other more traditional formats. PsycEXTRA is a high-quality, current, reliable resource for grey literature in the psychological and behavioral sciences.
